# Use of hospitals in the New York City Metropolitan Region, by race: how separate? How equal in resources and quality?

**DOI:** 10.1186/s12913-022-08414-3

**Published:** 2022-08-10

**Authors:** Bian Liu, Katherine A. Ornstein, Julia L. Frydman, Amy S. Kelley, Emma K. T. Benn, Albert L. Siu

**Affiliations:** 1 Department of Population Health Science and Policy, Icahn School of Medicine at Mount Sinai, New York, NY USA; 2 Brookdale Department of Geriatrics and Palliative Medicine, Icahn School of Medicine at Mount Sinai, 1 Gustave L. Levy Place, Box 1640, New York, NY 10029 USA; 3 Geriatric Research, Education, and Clinical Center, James J Peters Veterans Affairs Medical Center, Bronx, NY USA

**Keywords:** Disparities, Race and ethnicity, Health systems, Medicare, Dissimilarity index, Segregation

## Abstract

**Background:**

Although racial and ethnic minorities disproportionately use some hospitals, hospital-based racial and ethnic composition relative to geographic region and its association with quality indicators has not been systematically analyzed.

**Methods:**

We used four race and ethnicity categories: non-Hispanic white (NHW), non-Hispanic black (NHB), Hispanic, and Asian/Pacific Islander/Alaskan Native/American Indian (API/AIAN), as well as a combined non-NHW category, from the 2010 (latest year publicly available) Medicare Institutional Provider & Beneficiary Summary public use file for 84 hospitals in the New York City region. We assessed the relative distribution of race and ethnicity across hospitals grouped at different geographic levels (region, county, hospital referral region [HRR], or hospital service areas [HSA]) using the dissimilarity index. Hospital characteristics included quality star ratings, essential professional services and diagnostic/treatment equipment, bed size, total expenses, and patients with dual Medicare and Medicaid enrollment. We assessed Spearman’s rank correlation between hospital-based racial and ethnic composition and quality/structural measures.

**Results:**

Dissimilarity Index decreases from region (range 30.3–40.1%) to county (range 13.7–23.5%), HRR (range 10.5–27.5%), and HSA (range 12.0–16.9%) levels. Hospitals with larger non-NHW patients tended to have lower hospital ratings and higher proportions of dually-enrolled patients. They were also more likely to be safety net hospitals and non-federal governmental hospitals.

**Conclusions:**

In the NYC metropolitan region, there is considerable hospital-based racial and ethnic segregation of Medicare patients among non-NHW populations, extending previous research limited to NHB. Availability of data on racial and ethnic composition of hospitals should be made publicly available for researchers and consumers.

**Supplementary Information:**

The online version contains supplementary material available at 10.1186/s12913-022-08414-3.

## Introduction

Studies have shown that Black and Hispanic patients are disproportionately hospitalized in some hospitals and, in some cases, are highly concentrated in a smaller number of hospitals—thereby, creating de facto separate hospital care by race and ethnicity, or segregation [1–13]. Other work, primarily for Black patients, has shown that hospitals with a higher concentration of minority patients may have poorer performance on quality measures [14–16]. These hospitals may have systematic differences in organizational structure, revenue, staffing, services, and other resources.

Multiple interrelated factors may contribute to differences in patient volume and health outcomes across racial and ethnic groups. Patients may select a hospital due to proximity which, in turn, could be linked to residential segregation. Choice of hospitals may depend on the type and extent of health insurance coverage and that, in turn, could be linked to employment and income opportunities that may be influenced by discrimination and segregation. Patients and family may voluntarily choose a particular hospital due to language, culture, or religious affinity; [17, 18] however, even there, these hospitals may have been founded in response to discrimination and exclusion of patients or staff from other facilities [19]. In this sense, “segregation” may be an important contributor to the observation of “separate care.” For this reason, we will use the term “segregation” to describe “separate care.” This usage is consistent with how the term segregation is used to describe the similar phenomenon in housing and education [20–28].

Despite growing recognition of and interest in racial and ethnic segregation in US healthcare, the problem is not well studied due to numerous methodological challenges. First, race and ethnicity are inconsistently, unreliably, and variably recorded in administrative data, particularly for Hispanics [29–31]. These issues, however, could be potentially mitigated using different algorithms [32–34]. For example, an algorithm developed by the Research Triangle Institute (RTI) improves the accuracy of coding for Hispanic and Asian or Pacific Islander Medicare beneficiaries by 159% and 45%, respectively [29].

In addition, there exists a wide range of approaches to quantitatively describe racial and ethnic segregation with no consensus on what is the best measure and whether a single measure can capture its extent [20–23]. For example, “minority-serving hospitals,” a commonly used term in studying hospital health disparities, is an example of an absolute measure of segregation, as it is based on a threshold (e.g., 90^th^ or 75^th^ percentile) of the direct proportion of minority patients in a given hospital. The dissimilarity index (DI), a measure of segregation or imbalance or unevenness used in demography, housing, and education, is less often used in healthcare disparity research [24]. Moreover, in estimating the extent of segregation in healthcare, the measure of spatial resolution is not inconsequential. For example, variation of racial and ethnic composition across counties may differ from that measured across census tracts. This issue is known as a modifiable area unit problem, as the measures are influenced by the shape and scale of the aggregation unit [35]. Finally, there is a wide range of outcome variables for evaluating the impact of racial and ethnic segregation, ranging from health service utilization, mortality, and hospital quality, further complicating the approach to evaluation [12, 36].

In this analysis, we examine the extent of racial and ethnic segregation of Medicare patients seen in hospitals across various geographic levels in the New York City (NYC) metropolitan region. In addition, we examine the extent to which the racial and ethnic composition of a hospital is associated with hospital structural characteristics (e.g., bed size, access to essential services and technologies, and hospital expenditure) and a hospital’s publicly reported measures of quality. We focus on the NYC metropolitan region—the most populous and one of the more diverse and residentially segregated metropolitan regions in the Northeastern United States. By concentrating on one region, we can more accurately evaluate the impact of community size, the organization of the healthcare market, and effects of closures and consolidations (which may lead to 2 very different facilities reporting under one combined hospital identity) on hospital segregation.

## Methods

### Data sources

**Fig. 1:**
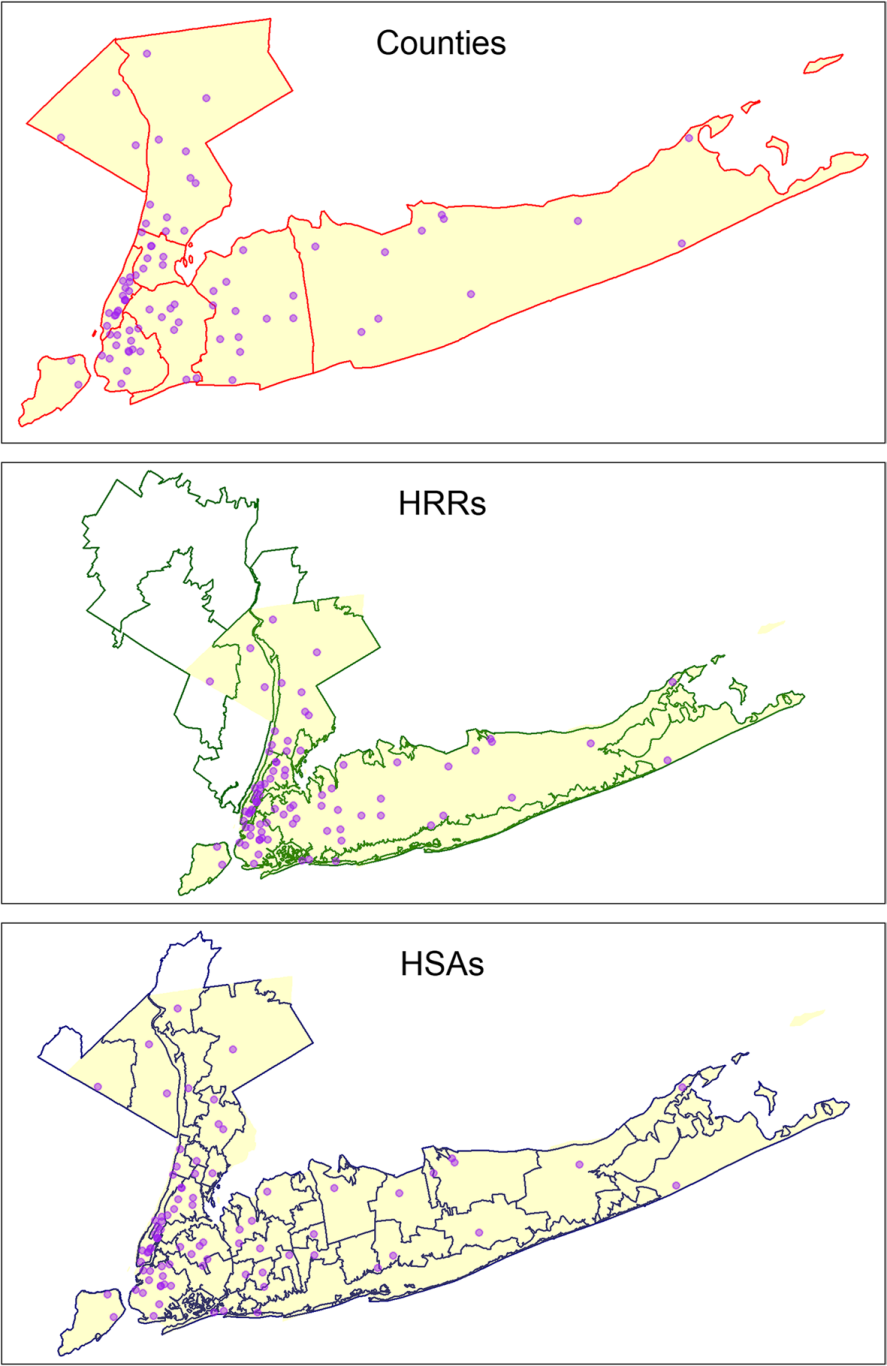
Locations of hospitals (dots) in New York City metropolitan area (shaded area) with different geographic boundaries: Counties, Health Referral Regions (HRRs), and Health Service Areas (HSAs)

Our analyses used data from multiple public use files (PUFs) from the Centers for Medicare and Medicaid Services (CMS). Hospital-level information on patient race and ethnicity was obtained from the 2010 (latest year publicly available) Institutional Provider and Beneficiary Summary (IPBS) PUF, which included 100% claims for the Medicare fee-for-service (FFS) population from hospitals and other institution types (*n* = 49,413) [37]. We also obtained the 2021 overall hospital quality star ratings from the Hospital General Information file provided by CMS (*n* = 5382) [38]. We used data from 2021 to address concerns over the previous methodology in use in 2010 and due to inaccessibility of the earlier data [39–41]. Information related to the hospital’s hospital referral regions (HRRs) and hospital service areas (HSAs) was obtained from the Dartmouth Atlas Project for the year 2010 (*n* = 4893) [42]. HSAs are essentially a group of ZIP codes that represent local markets for hospital care, and many HSAs contain only one 1 or a few number of hospitals. HRRs represent regional markets for tertiary care and contain at least one hospital performing major cardiovascular or neurosurgical procedures [43]. In addition, hospital structural characteristics (e.g., bed size, access to essential services and technologies, and hospital expenditure) were obtained from the American Hospital Association (AHA) annual survey for the year 2010 (*n* = 5915) [44]. We identified hospitals existed in all sources (*n* = 4893), while excluding hospitals located in the US territories (*n* = 79). We further limited analyses to 84 hospitals within the NYC metropolitan region that covers 9 counties, 6 HRRs, and 37 HSAs (Fig. 1). These included partial HRRs and HSAs as our study area of interest was the 9 counties within the state of New York (Supplemental Fig. S1).

## Measures

### Hospital racial/ethnic composition

The IPBS PUF summarized the number of beneficiaries who utilized services (e.g., stays, admissions, visits, episodes) of the institutional provider, and the numbers of beneficiaries for the following five groups using the enhanced race and ethnicity designation according to the RTI race code. We report on 4 race and ethnic categories both separately and combined: non-Hispanic White (NHW), non-Hispanic Black (NHB), Hispanic, Asian/Pacific Islander, Alaskan Native/American Indian (API/AIAN), and a combined category designated as minorities which includes the NHB, Hispanic, API/AIAN and the small (< 1% of all beneficiaries on average) categorized as “other” in the RTI race code and who would otherwise be excluded from the analysis due to sample size issues; this category, in effect, includes all groups that are not NHW (non-NHW). We calculated the proportion of each group by dividing the number of beneficiaries in each subgroup by the total number of beneficiaries. Subgroups with beneficiary numbers less than 11 are suppressed in the public data release to protect patient privacy. A hospital could have one or more subgroups with missing beneficiary counts. We calculated the difference between the total beneficiary counts and the sum of all available race and ethnicity subgroups, and divided this difference by the total number of subgroups with missing data to replace the missing number beneficiary counts.

### Dissimilarity index

We used DI to assess the evenness of the distribution of racial and ethnic group across hospitals relative to the overall distribution of that racial and ethnic group in the hospital’s geography, similar to the method used for studying the US public school system [24]. For a particular racial/ethnic group *x* in a geographic area of *G*, ${DI}_{x}$ is derived according to the following equation: ${DI}_{x}=\sum_{i=1}^{n}\frac{{p}_{i}|{m}_{i}-M|}{2PM(1-M)}$, where ${p}_{i}$ is the number of patients in hospital *i*, P is the number of patients in area *G*, ${m}_{i}$ is the percentage of patients of racial/ethnic group *x* in hospital *i*, M is the percentage of patients of racial/ethnic *x* in area *G*, and area *G* has *n* hospitals (*i* = *1, 2,…n*). We calculated DI for each racial/ethnic group (i.e., *x* = *(NHW, NHB, Hispanic, API/AIAN, or Minorities)*) at four different geographic levels (i.e., *G* = *(NYC metropolitan area, counties, HRRs, or HSAs)*).

### Hospital characteristics

We included the following eight hospital characteristics. The CMS 5-start hospital overall rating is a composite measure of hospital quality based on individual indicators from five domains: mortality, safety of care, readmission, patient experience, and timely and effective care [38]. The rating is on a 0–5 scale with 5 being the highest rating, and was calculated according to the updated algorithm. From the AHA data on hospital services, we surveyed 5 physicians to identify services that would be most essential in the care of an adult medical/surgical patient in a community hospital (excluding services that could be appropriately performed elsewhere after discharge or if the patient could be reasonably transferred to a tertiary hospital). From their consensus ratings, we defined “essential professional services” as the total number of the following 7 services: Emergency Department, Adult cardiology services, Neurological services, Oncology services, Orthopedic services, Psychiatric child/adolescent services, and Palliative Care Program. We also defined “essential diagnostic, treatment and imaging” as the total number of the following 9 items: Adult diagnostic catheterization, Adult interventional cardiac catheterization, Adult cardiac electrophysiology, Hemodialysis, Optical Colonoscopy, Endoscopic ultrasound, Computed-tomography (CT) scanner, Magnetic resonance imaging (MRI), and Ultrasound. In addition, we calculated the relative proportion of ICU beds to total general beds, the total expense per hospital bed, and ownership (non-federal government (*n* = 16) or non-profit (*n* = 68)) [44]. We also included the proportion of patients with dual Medicare and Medicaid enrollment from the IPBS PUF as an indicator of socioeconomic disadvantage. Finally, according to the Agency for Healthcare Research and Quality (AHRQ) definition, we identified safety net hospital (*n* = 32) based on top quartile of hospitals ranked by the percentage of total discharges that are composed of uninsured patients or those with Medicaid [45].

### Statistical analysis

We summarized patient racial and ethnic composition using box-plots. We reported the median and interquartile range (IQR) of DI for each race and ethnicity group at four geographic levels. We also reported the Spearman’s rank correlation coefficients between specific hospital-level racial/ethnic compositions (e.g., percent NHW) and hospital characteristics. We also compared hospital race/ethnicity composition and characteristics by ownership and safety net status using Wilcoxon rank-sum tests. The analyses were conducted using R (v4.0.2) with RStudio (v1.3.1073).

## Results

For the entire NYC metropolitan area, the proportion of patients who were NHW, NHB, Hispanic, AAPI/AIAN, and minorities was 66.6%, 15.1%, 13.0%, 3.7%, and 33.4%, respectively. Racial and ethnic compositions at county-, HRR-, and HSA-level are shown in Fig. S2 in the Supplementary Information, and hospital characteristics at county-level are shown in Table 1. For the entire NYC metropolitan area, the DIs for NHW, NHB, Hispanic, and API/AIAN were 40.1%, 36.1%, 37.6%, and 30.3%, respectively (Table 2). A regional DI of 40.1% can be interpreted as the share of NHW patients (or combined minority patients) that would need to change hospitals to create a perfectly integrated region-wide hospital system. The range of median DI diminished to between 13.7% and 23.5% at the county level, between 10.5% to 27.5% at the HRR level, and between 12% and 16.9% at the HSA level (Table 2).

**Table 1:** Summary descriptive statistics of hospital characteristics at county level

Variables		Bronx (*n* = 8)	Kings (*n* = 14)	Nassau (*n* = 11)	New York (*n* = 15)	Queens (*n* = 9)	Richmond (*n* = 2)	Rockland (*n* = 3)	Suffolk (*n* = 11)	Westchester (*n* = 11)
**Race/Ethnicity composition**	**Non-Hispanic White**	19.2 (13.7)	34.5 (26)	80.4 (11.4)	49.5 (27)	47.3 (17)	76 (11.1)	83.2 (4.1)	88.3 (5.9)	73.6 (18)
	**Non-Hispanic Black**	28.6 (6.9)	40.5 (27.8)	11.5 (9.4)	22.4 (20.3)	23.1 (13.4)	9.7 (6.5)	8.2 (2.6)	5.3 (3)	15.8 (15.5)
	**Hispanic**	42.7 (15.9)	18.5 (15)	4.8 (1.6)	20.3 (12.3)	16.7 (8.3)	9.5 (4.3)	5.5 (1.2)	4.5 (3.1)	7.6 (5.6)
	**Asian/Pacific Islander, Alaskan Native/American Indian**	6.1 (11)	3.3 (2.5)	2.2 (1)	6.4 (8.9)	9.6 (6.5)	3.2 (1.4)	2.4 (0.5)	1.2 (0.4)	2.1 (0.6)
	**Minorities**	80.8 (13.7)	65.5 (26)	19.6 (11.4)	50.5 (27)	52.7 (17)	24 (11.1)	16.8 (4.1)	11.7 (5.9)	26.4 (18)
**Hospital characteristics**	**Overall hospital ranking**	1.3 (0.5)	1.4 (0.7)	2.4 (1.7)	3.3 (1.4)	1.7 (1.3)	1.5 (0.7)	2 (0)	2.9 (1.4)	2.4 (1.1)
	**ICU%**	15.5 (7.6)	15 (4)	15.6 (11.2)	13 (8.4)	12.1 (4.1)	17.9 (0.3)	10.3 (14.5)	14.4 (4)	11.8 (4.9)
	**Total expenses ($) per hospital bed**	990,736 (516,319)	1,003,784 (296,045)	916,764 (475,734)	1,884,923 (1,058,949)	935,901 (272,269)	906,971 (244,789)	647,691 (166,496)	899,542 (297,737)	746,995 (313,839)
	**Number of essential professional services**	6.2 (1)	6.1 (0.9)	6.2 (0.9)	5.8 (2.1)	5.6 (1.1)	6 (0)	2.5 (0.7)	5.8 (1.1)	5.3 (2.3)
	**Number of essential diagnostic, treatment and imaging**	6.2 (1.9)	7.2 (1.4)	7.5 (1.4)	6.8 (2.4)	6 (2.3)	7 (0)	2.5 (2.1)	6.4 (1.7)	5.2 (2.6)
	**Proportion of patients with dual Medicare Medicaid enrollment**	74.6 (16.1)	69.5 (9.6)	24.3 (10.1)	47.8 (24.3)	60.8 (15.8)	42.9 (10)	23.1 (6)	20.9 (7.4)	27.5 (15.8)
	**Number of top safety net hospitals**	6 (75%)	10 (71%)	1 (9%)	5 (33%)	4 (44%)	1 (50%)	1 (33%)	0 (0%)	4 (36%)
	**Number of non-federal government hospitals**	3 (38%)	4 (29%)	1 (9%)	3 (20%)	2 (22%)	0 (0%)	1 (33%)	1 (9%)	1 (9%)
	**Number of non-profit hospitals**	5 (63%)	10 (71%)	10 (91%)	12 (80%)	7 (78%)	2 (100%)	2 (67%)	10 (91%)	10 (91%)

Minorities are the sum of non-Hispanic Black, Hispanics, Asian/Pacific Islander, Alaskan Native/American Indian, and other race/ethnicity groups that are not non-Hispanic White. The sum of NHW and minorities equals to one

“Essential professional services” is defined as the total number of the following 7 services: Emergency Department, Adult cardiology services, Neurological services, Oncology services, Orthopedic services, Psychiatric child/adolescent services, and Palliative Care Program. “Essential diagnostic, treatment and imaging” was defined as the total number of the following 9 items: Adult diagnostic catheterization, Adult interventional cardiac catheterization, Adult cardiac electrophysiology, Hemodialysis, Optical Colonoscopy, Endoscopic ultrasound, Computed-tomography (CT) scanner, Magnetic resonance imaging (MRI), and Ultrasound

**Table 2:** Distribution of the Dissimilarity Index (median (interquartile range, IQR)) by racial/ethnic group by geographic level, New York City metropolitan area, 2010

Geographic level	Non-Hispanic White	Non-Hispanic Black	Hispanic	Asian/Pacific Islander, Alaskan Native/American Indian
NYC metropolitan region (*n* = 1)	40.1	36.1	37.6	30.3
Counties (*n* = 9)	23.5 (18.1–25.7)	23.5 (20.9–26.8)	20.2 (14.2–21.5)	13.7 (11.2–22.3)
HRRs (*n* = 6)	27.5 (14.5–32.1)	18.2 (8.1–29.6)	23.7 (11.9–25.9)	10.5 (8.6–20.2)
HSAs (*n* = 37)	16.9 (11.5–21.2)	13.4 (4.6–17.9)	13.6 (8.5–20.5)	12.0 (8.3–13.8)

Minorities are the sum of non-Hispanic Black, Hispanics, Asian/Pacific Islander, Alaskan Native/American Indian, and other race/ethnicity groups that are not non-Hispanic White. Due to the symmetry between NHW and minorities (the sum of these two proportion equals to one) in calculating DI, measures of DI for NHW is the same as DI for minorities

**Fig. 2:**
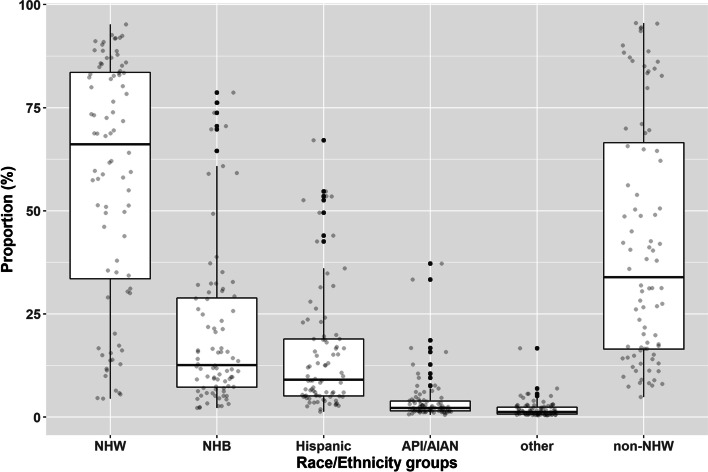
Distributions of patient racial and ethnic compositions among 84 hospitals in the New York City metropolitan area. Note: NHW = non-Hispanic White, NHB = non-Hispanic Black, API/AIA*N* = Asian/Pacific Islander, Alaskan Native/American Indian, non-NHW = a combined category designated as minorities which includes the NHB, Hispanic, API/AIAN and the small (< 1% of all beneficiaries on average) categorized as “other” in the RTI race code

Across 84 hospitals in the NYC metropolitan area, the median percentage of patients who were NHW, NHB, Hispanic, API/AIAN, and racial/ethnic minorities was 66.1% (IQR 33.5%-83.5%), 12.6% (IQR 7.3%-28.9%), 9.1% (IQR 5.1%-28.9%), and 2.2% (IQR 1.5%-3.9%), respectively (Fig. 2).

The proportion of NHB was closely correlated with the proportion of Hispanic (Spearman’s correlation coefficient (*ρ*) = 0.73, *p* < 0.0001), while not significantly correlated with the proportion of API/AIAN (*ρ* = 0.15, *p* = 0.18). The proportion of NHW patients was significantly inversely correlated with other groups (Table 3). The racial and ethnic distribution of patients varied at HRR, county, and HSA levels. For example, the percentage of minority patients ranged from 7.4 to 26.1% in Suffolk county, 8.2 to 41.2% in Nassau county, 56.2 to 95.5% in the Bronx, 23.6 to 94.5% in Brooklyn, 38.3 to 83.8% in Queens, 15.1 to 95.4 in Manhattan, and 7.6 to 68.9% in Westchester county (see Table S1). Two hospitals a short distance apart could have marked differences in the percentage of NHBs (e.g., 23.3 vs 7.0% in the same part of Manhattan) or Hispanics (e.g., 36.1 vs. 8.3% in the same part of Manhattan) (Table S1).

**Table 3:** Correlations of race specific percentages at level of individual hospitals (*n* = 84) in New York City metropolitan area

	**Non-Hispanic Black**	**Hispanic**	**Asian/Pacific Islander, Alaskan Native/American Indian**	**Minorities**
Non-Hispanic White	-0.93 (*p* < 0.0001)	-0.87 (*p* < 0.0001)	-0.29 (*p* = 0.008)	-1.00 (*p* < 0.0001)
Non-Hispanic Black		0.73 (*p* < 0.0001)	0.15 (*p* = 0.18)	0.93 (*p* < 0.0001)
Hispanic			0.41 (*p* = 0.0001)	0.87 (*p* < 0.0001)
Asian/Pacific Islander, Alaskan Native/American Indian				0.29 (*p* = 0.007)

Minorities (i.e. non-NHW) includes non-Hispanic Black, Hispanic, Asian/Pacific Islander, Alaskan Native/American Indian, and other race/ethnicity groups

The proportion of NHB, Hispanic, API/AIAN, and combined racial/ethnic minority groups were all positively correlated with the proportion of patients with dual enrollment in Medicare and Medicaid (ρ ranged from 0.27 to 0.91, all *p* < 0.0001, Table 4), while the opposite was found for NHW (ρ = -0.91, *p* < 0.0001). Indicators of hospital structure/resources were in general not correlated with patient racial and ethnic composition (Table 4).

**Table 4:** Spearman’s correlation coefficients between proportion of race and ethnicity groups and measures of structure and quality of the hospital

	**Overall hospital ranking** (*n* = 65)	**ICU%** (*n* = 70)	**Total expenses per hospital bed** (*n* = 81)	**Essential professional services** (*n* = 71)	**Essential diagnostic, treatment and imaging** (*n* = 71)	**Proportion of patients with dual Medicare Medicaid enrollment**
Minorities%	-0.55 (*p* < 0.001)	0.06 (*p* = 0.62)	0.07 (*p* = 0.51)	0.13 (*p* = 26)	0.03 (*p* = 0.82)	0.91 (*p* < 0.0001)
Non-Hispanic Black	-0.57 (*p* < 0.0001)	0.01 (*p* = 0.92)	-0.04 (*p* = 0.71)	0.16 (*p* = 0.19)	0.05 (*p* = 0.67)	0.80 (*p* < 0.0001)
Hispanic	-0.40 (*p* = 0.001)	0.06 (*p* = 0.63)	0.12 (*p* = 0.27)	0.18 (*p* = 0.14)	0.06 (*p* = 0.61)	0.84 (*p* < 0.0001)
Asian/Pacific Islander, Alaskan Native/American Indian	0.01 (*p* = 0.96)	0.10 (*p* = 0.43)	0.31 (*p* = 0.005)	0.10 (*p* = 0.39)	0.13 (*p* = 0.27)	0.27 (*p* < 0.0001)
Non-Hispanic White	0.55 (*p* < 0.0001)	-0.06 (*p* = 0.62)	-0.07 (*p* = 0.51)	-0.13 (*p* = 26)	-0.03 (*p* = 0.82)	-0.91 (*p* < 0.0001)

Minorities are the sum of non-Hispanic Black, Hispanics, Asian/Pacific Islander, Alaskan Native/American Indian, and other race/ethnicity groups that are not non-Hispanic White. The sum of NHW and minorities equals to one

“Essential professional services” is defined as the total number of the following 7 services: Emergency Department, Adult cardiology services, Neurological services, Oncology services, Orthopedic services, Psychiatric child/adolescent services, and Palliative Care Program. “Essential diagnostic, treatment and imaging” was defined as the total number of the following 9 items: Adult diagnostic catheterization, Adult interventional cardiac catheterization, Adult cardiac electrophysiology, Hemodialysis, Optical Colonoscopy, Endoscopic ultrasound, Computed-tomography (CT) scanner, Magnetic resonance imaging (MRI), and Ultrasound

The proportion of NHB and Hispanic was negatively correlated with the overall hospital rating (*ρ* = -0.57 and -0.40, respectively, both *p* < 0.0001, Table 4), while the proportion of NHW patients was significantly positively correlated with the overall hospital rating (*ρ* = 0.55, *p* < 0.0001). The correlation between hospital rating and the proportion of API/AIAN was not significant (*ρ* = 0.01, *p* = 0.96). Safety net hospitals had a higher proportion of racial/ethnic minority patients or dual eligible patients, and lower overall hospital rating (Figure S3). Similar trends were seen in non-federal governmental hospitals (Figure S4).

## Discussion

We found considerable racial and ethnic segregation among Medicare patients in hospitals in the NYC region. The segregation was present within county, HRR, and HSA geographic levels, although the unevenness of racial and ethnic distribution became progressively (and expectedly) less at each level. This segregation at the geographic level was accompanied by examples of more extreme disparities in racial and ethnic composition between some hospitals. Our analyses indicate that the uneven racial and ethnic composition at the hospital level was weakly associated with structural measures of healthcare service capacity but more strongly associated with publicly reported hospital quality rankings, safety net hospital status, and hospital ownership type.

These findings are consistent with previous work that has shown that there is racial segregation in where patients receive hospital care in the United States [7–13, 46]. The majority of that research has focused on segregation of the Black population. Moreover, these findings similarly find segregation associated with lower hospital quality [1–6, 47]. The current analysis adds to this literature by quantifying the extent of racial and ethnic segregation using a validated algorithm to classify race from administrative data [29]. These findings demonstrate that the association of these disparate racial distributions with measures of hospital quality extend beyond Black populations to other racial/ethnic minority group populations. We also build upon previous research by demonstrating the extent of racial and ethnic segregation in the most populous and one of the most ethnically and racially diverse metropolitan regions in the United States. Ongoing questions about the existence of structural racism throughout American medicine including within its most high profile publications underlie the importance of further quantifying and illustrating the extent of hospital segregation [48].

The dissimilarity we report is slightly lower than the levels of dissimilarity reported for residential communities, but similar to school segregation in the US [24, 25]. Nationally, residential segregation in metropolitan areas has decreased from DIs of 72.7 in 1980 to 55.2 in 2020 for the Black-White DI, from 50.2 to 45.3 for the Hispanic-White DI, and from 40.4 to 40.0 for the Asian-White DI [26]. In the New York-Jersey-City-White Plains metropolitan areas, higher residential segregation was found ranging from DIs of 79.1 for Black-White, 63.1 for Hispanic-White, and 49.5 for Asian-White in 2010 [25]. Public school segregation for Black and Hispanic students was about 32% nationally and 55% in metropolitan areas between 1995 and 2015 [24]. While similar to trends in public school segregation, the DI values we report decreased with smaller geographic units, disparities remain even at the finest geographic levels. Smaller geographic divisions may be more homogenous in race and ethnicity either due to segregationist policies or preferences (e.g., a predominantly ethnic community), and examining dissimilarity at different geographic levels is important to understanding its context. It is not surprising that healthcare is highly associated with where people reside and suggests that many of the underlying causes of residential, school, and hospital segregation are pervasive and shared.

Some of the underlying causes of segregation in healthcare that are not encountered in housing and school segregation are worth noting. In particular, the United States maintains a system of health insurance with eligibility conditioned on employer, age, income, service in the armed forces, and other factors. The design of insurance products (with payments of varying generosity, preferred networks, and physician participation or non-participation) provides further opportunity for sorting by income and race [49, 50]. For example, unlike hospitals, a small percentage of physicians choose not to participate in Medicare and a larger number place limits on new patients due to payment levels or other conditions; many more physicians limit Medicaid participation [51, 52]. Existing patient-physician relationships and physician-hospital affiliations will heavily influence where patients will be referred for hospitalization. Some patients will have no choice but to rely on safety net hospitals [53, 54]. Indeed, we found that AHRQ-definition safety-net hospitals had a higher median percentage of racial/ethnic minority Medicare patients [45, 55]. Longstanding provider selections made under limited coverage options may continue even after more choices become available through Medicare eligibility. Apart from features of the insurance market, patients may also intentionally choose healthcare settings due to geographic proximity, language, cultural or religious considerations [56, 57]. However, it is important to note that some of these healthcare organizations were created in response to exclusion and discrimination against specific patients and/or hospital staff such as physicians of specific races, ethnicities, or religions. As a result, the organization of healthcare and the structure of health insurance may lead to systemic segregation, which may affect where receive care, what resources will be available to treat them and, in turn, affect healthcare quality and patient outcomes. This healthcare landscape would suggest that insurance coverage of the population, including all-payor hospital rate regulation approaches such as that in Maryland, focused efforts to improve quality at low-performing hospitals, and public reporting of race and ethnicity by hospitals and health plans may be important and needed tools in a multifaceted approach to reduce and correct health facility segregation [14, 58, 59].

Our analysis has some limitations. We focused on segregation that might be observed between hospitals, and segregation that might occur within a facility is beyond the scope of our data. The race and ethnicity variable was obtained from Medicare fee-for-service data from 2010, and the quality outcome variables were obtained from the 2021 CMS Hospital General Information file.–We used the 2010 Medicare data because it was the latest hospital-level public use file available with the RTI classification of patient race and ethnicity. Ideally, we would have used quality ratings more proximate in time to 2010, but the methods of the hospital overall quality rating have been updated over time to overcome criticism of older methods. We used the more recent quality ratings because we found that the relative rating of the hospitals (*n* = 58 available for all 6 years) to be relatively stable in analyses of quality ratings from 2015–2020 (see Fig S5). This approach was carried out to provide the research team with the opportunity to test the use and limitations of these data to justify the cost and privacy safeguards necessary to perform the research with more recent all-payor datasets. Nevertheless, our analysis involves Medicare – the largest single payor in the United States. Although more recent data would be useful, recent data on the experience of New York area public and private hospitals during the SARS-CoV-2 pandemic would suggest that the racial and ethnic distributions of these hospitals have not substantively changed [60].

Furthermore, our analysis was limited to the NYC metropolitan area, thereby potentially limiting its generalizability. Our approach sought to better understand the data on a more granular level than would have been possible with a national focus. By focusing on the regional level, we were able to understand why no data (due to hospital consolidation) was available for some hospitals that were known to exist, why only limited data existed for some hospitals (due to closures), and why selected data elements were not available for some hospitals (due to exemption from reporting). Our nuanced, local understanding gave the research team greater confidence in the data, as well as an understanding of its limitations. Future research should consider how the consolidation of hospitals may potentially reduce and obscure differences in the racial and ethnic distribution of the unmerged entities.

Finally, our research relied on the RTI classification of race and ethnicity using data that is not consistently self-reported, may misclassify some individuals, and may aggregate some categories (e.g., among Hispanics or the API/AIAN category) [61]. Nonetheless, we believe this analysis advances previous research that may have used race and ethnicity variables that are often inconsistently and unreliably recorded in administrative data. Understanding the experience of smaller racial and ethnic subgroups is critical and may require other types of data and research approaches.

## Conclusions

We report that segregation of Medicare patients in NYC metropolitan areas hospitals is pervasive, and associated with disparities in measures of hospital quality. Segregation was observed across NHB, Hispanic, and API/AIAN groups. The organization of healthcare and of health insurance may lead to systemic segregation, which may affect where patients receive care, which hospitals are used, which doctors are seen, what resources will be available to treat their conditions, and the outcomes of care. Additional research is needed with updated data, inclusive of other payors, and with a national focus to further our understanding of how care is segregated and how best to advance equity in health services for the American people.

## Supplementary Information

**Additional file 1: Fig. S1.** The locations of hospitals (dots) in New York City metropolitan area (shaded area) with different geographic boundaries: Counties (red outlines), Health Referral Regions (HRRs; green outlines), and Health Service Areas (HSAs; blue outlines). **Fig. S2.** Distributions of patient racial/ethnic compositions at different level of geography level in the New York City metropolitan area. CNTY=county, HRR=health referral region, HSA=health service area. **Fig. S3.** Distributions of patient racial/ethnic compositions and hospital characteristics by top safety net hospital status. **Fig. S4.** Distributions of patient racial/ethnic compositions and hospital characteristics by hospital ownership. **Fig. S5.** Distribution of hospital overall rating during the years 2015-2020. **Table S1.** Proportion of patient race/ethnicity across 84 hospitals in the New York City Metropolitan area. (Minorities are the sum of non-Hispanic Black, Hispanics, Asian/Pacific Islander, Alaskan Native/American Indian, and other race/ethnicity groups that are not non-Hispanic White).

## Data Availability

The study used several data sets from the public domain, including 1) the 2010 (latest year publicly available) Institutional Provider and Beneficiary Summary (IPBS) public use files PUF from the Centers for Medicare and Medicaid Services (CMS), available from https://www.cms.gov/Research-Statistics-Data-and-Systems/Downloadable-Public-Use-Files/BSAPUFS/IPBS_PUF; 2) the 2021 overall hospital quality star ratings from the Hospital General Information file provided by CMS, available from https://data.cms.gov/provider-data/topics/hospitals/overall-hospital-quality-star-rating/; 3) the Dartmouth Atlas Project for the year 2010, available from https://data.dartmouthatlas.org/supplemental/#hospital, for information related to the hospital’s HRRs and HSAs; and 4) the American Hospital Association annual survey for the year 2010, available from https://www.ahadata.com/aha-annual-survey-database, for the following hospital-level information about hospital structural characteristics (e.g., bed size, access to essential services and technologies, and hospital expenditure). The datasets used and/or analyzed during the current study available from the corresponding author on reasonable request.
