# Supplementary material for: Use of hospitals in the New York City Metropolitan Region, by race: how separate? How equal in resources and quality?
Source: BMC Health Serv Res. 2022 Aug 10;22:1021. doi: 10.1186/s12913-022-08414-3 (PMC9365444; doi:10.1186/s12913-022-08414-3)
Supplement: Supplementary file 1 — Additional file 1: Fig. S1. The locations of hospitals (dots) in New York City metropolitan area (shaded area) with different geographic boundaries: Counties (red outlines), Health Referral Regions (HRRs; green outlines), and Health Service Areas (HSAs; blue outlines). Fig. S2. Distributions of patient racial/ethnic compositions at different level of geography level in the New York City metropolitan area. CNTY=county, HRR=health referral region, HSA=health service area. Fig. S3. Distributions of patient racial/ethnic compositions and hospital characteristics by top safety net hospital status. Fig. S4. Distributions of patient racial/ethnic compositions and hospital characteristics by hospital ownership. Fig. S5. Distribution of hospital overall rating during the years 2015-2020. Table S1. Proportion of patient race/ethnicity across 84 hospitals in the New York City Metropolitan area. (Minorities are the sum of non-Hispanic Black, Hispanics, Asian/Pacific Islander, Alaskan Native/American Indian, and other race/ethnicity groups that are not non-Hispanic White). [file 12913_2022_8414_MOESM1_ESM.docx]

**Use of Hospitals in the New York City Metropolitan Region, by Race: How Separate? How Equal in Resources and Quality?**

**Supplemental Information**

**Fig. S1.** The locations of hospitals (dots) in New York City metropolitan area (shaded area) with different geographic boundaries: Counties (red outlines), Health Referral Regions (HRRs; green outlines), and Health Service Areas (HSAs; blue outlines).

**Fig. S2.** Distributions of patient racial/ethnic compositions at different level of geography level in the New York City metropolitan area. CNTY=county, HRR=health referral region, HSA=health service area.

**Fig. S3.** Distributions of patient racial/ethnic compositions and hospital characteristics by top safety net hospital status.

**Fig. S4.** Distributions of patient racial/ethnic compositions and hospital characteristics by hospital ownership.

**Fig. S5.** Distribution of hospital overall rating during the years 2015-2020.

**Table S1.** Proportion of patient race/ethnicity across 84 hospitals in the New York City Metropolitan area. (Minorities are the sum of non-Hispanic Black, Hispanics, Asian/Pacific Islander, Alaskan Native/American Indian, and other race/ethnicity groups that are not non-Hispanic White.)

**Fig. S1.** The locations of hospitals (dots) in New York City metropolitan area (shaded area) with different geographic boundaries: Counties (red outlines), Health Referral Regions (HRRs; green outlines), and Health Service Areas (HSAs; blue outlines).


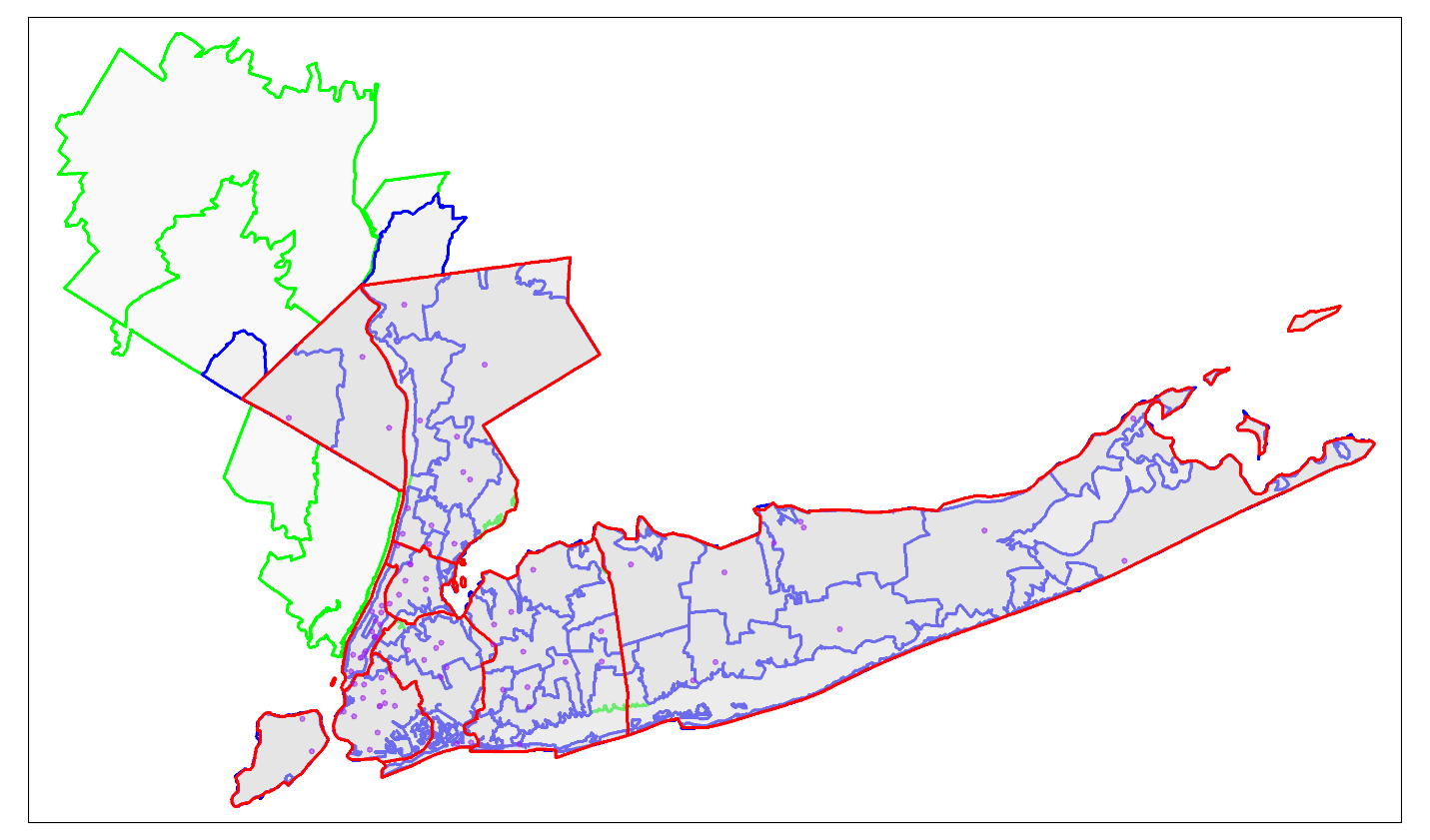


**Fig. S2.** Distributions of patient racial/ethnic compositions at different level of geography level in the New York City metropolitan area. CNTY=county, HRR=health referral region, HSA=health service area.


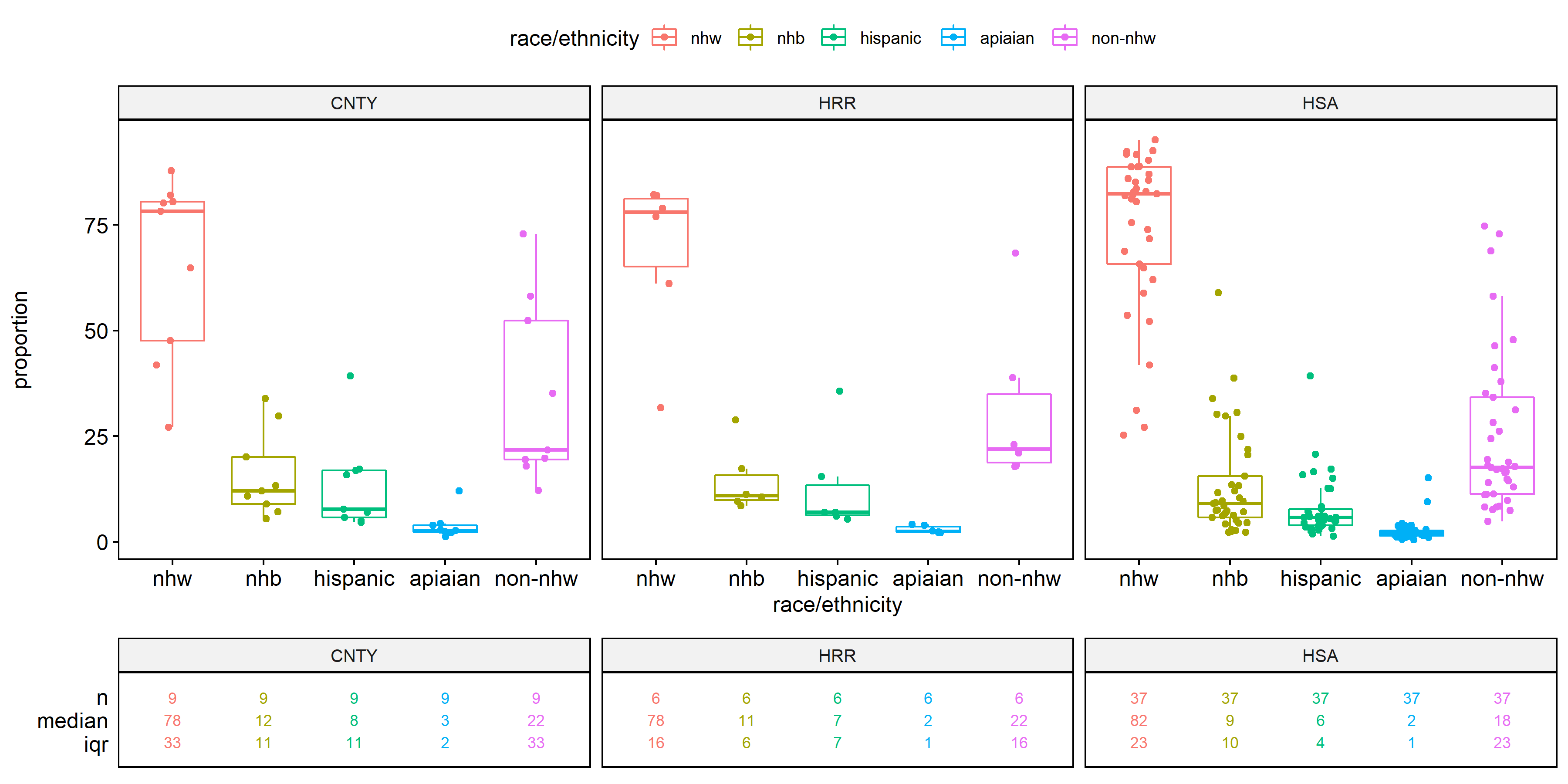


**Fig. S3.** Distributions of patient racial/ethnic compositions and hospital characteristics by top safety net hospital status. We defined top safety net hospital (n=32 in our sample) as within the top quartile of hospitals ranked by the percentage of the hospital’s discharges that are Medicaid and uninsured patients according to the following references: https://nyshealthfoundation.org/wp-content/uploads/2018/01/new-york-state-patients-safety-net-hospitals-jan-2018.pdf.


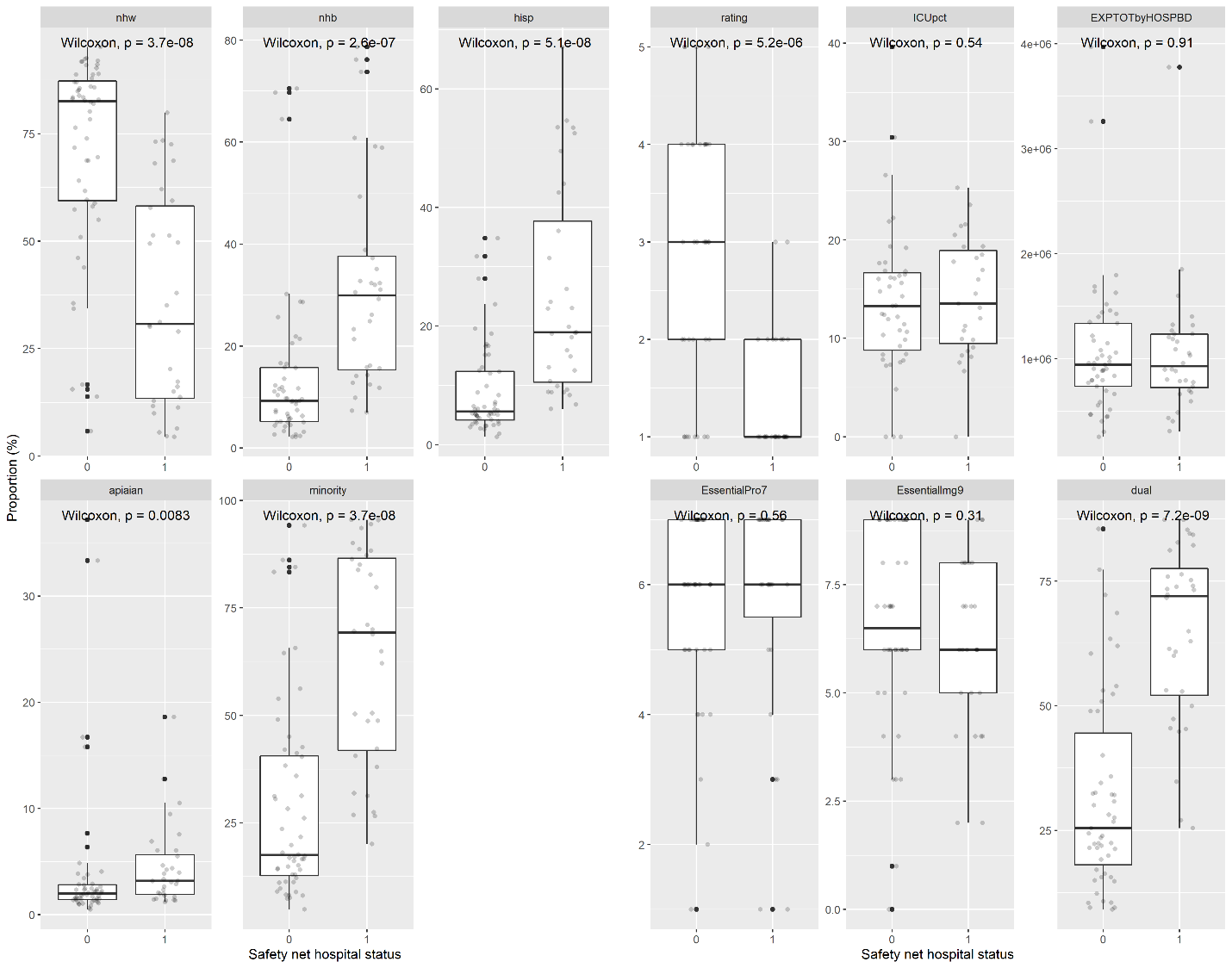


**Fig. S4.** Distributions of patient racial/ethnic compositions and hospital characteristics by hospital ownership.


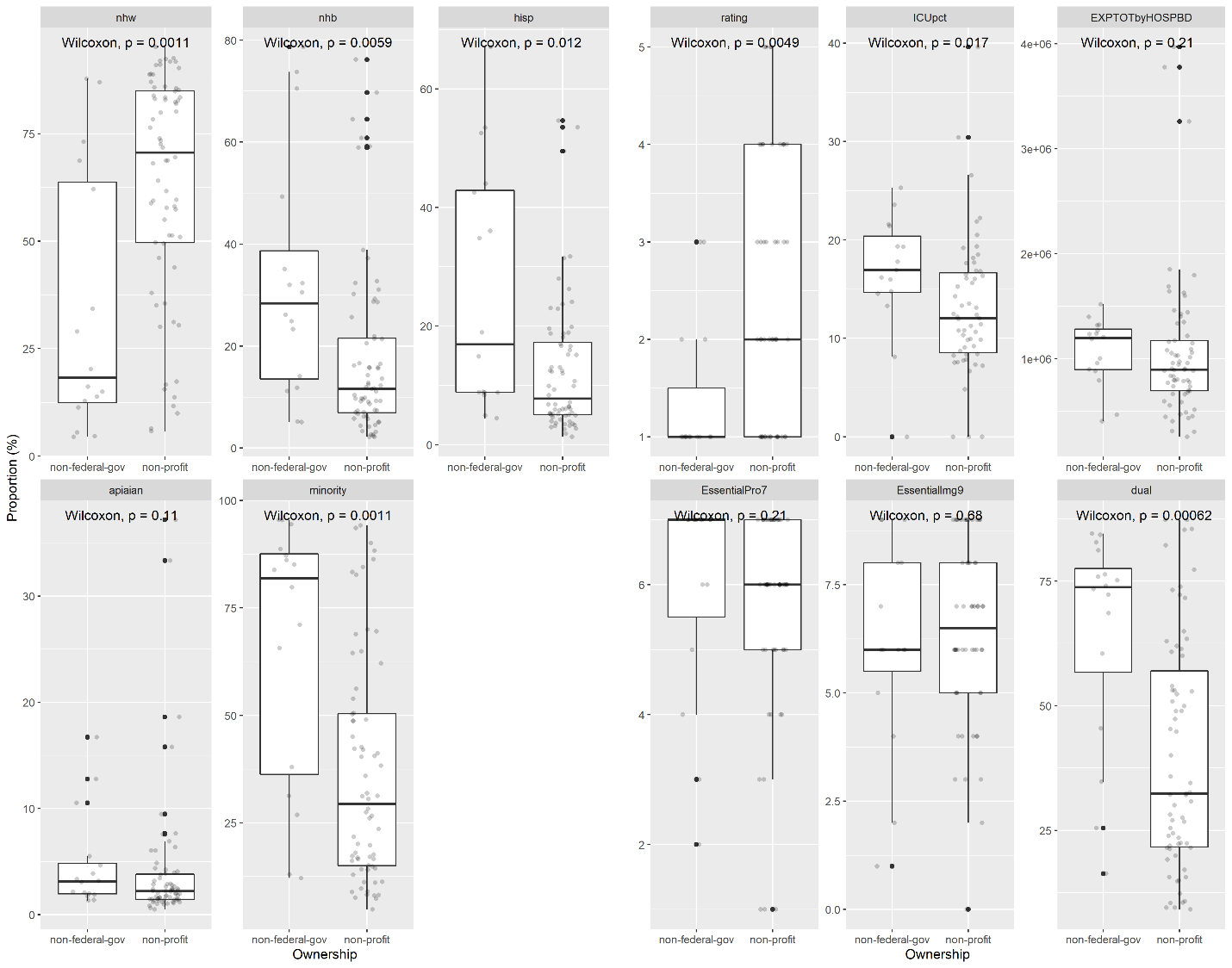


In terms of the hospital quality data, there are a few reasons we chose the 2020 data. First, the methods of hospital overall rating have been updated recently to overcome some of the criticism on older methods. We think the updated methods provide a better comparison across hospitals. Second, per CMS website, only data from the last 7 years were available (<https://data.cms.gov/provider-data/archived-data/hospitals>). While 2010 data were listed in the archived page, the file was not downloadable (<https://wayback.archive-it.org/org-551/20160104125435/https://data.medicare.gov/data/archives/hospital-compare>). We are unfortunately left with more recent data. Third, we did an additional analysis using the publicly available data for years 2015-2020, and found the relative rating of the hospitals (n=58 available for all 6 years) to be relatively stable (see Fig. S5 below).

**Fig. S5.** Distribution of hospital overall rating during the years 2015-2020.


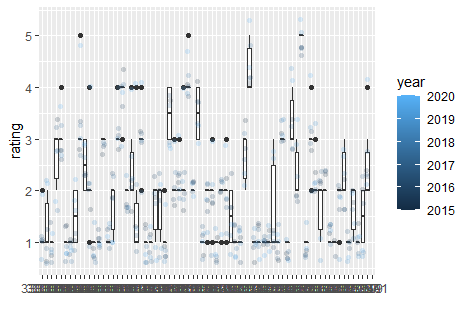

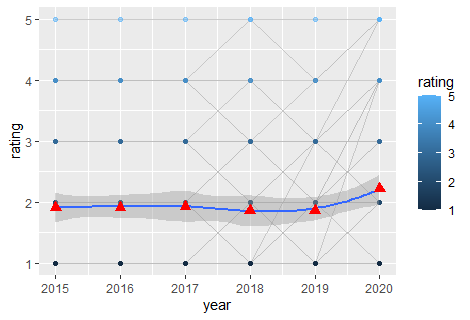
The red triangles in the Figure above shows the average rating (grey band indicates 95%CI) for all hospitals. The grey lines indicate individual hospital rating change over time. The figure indicate most hospitals remained their relative rating. A few hospitals experienced rating changes. However the changes are mostly limited to adjacent rankings (e.g., move up or down by 1), as shown in the Box Plot (where the axis is for individual hospitals).

**Table S1.** Proportion of patient race/ethnicity across 84 hospitals in the New York City Metropolitan area. (Minorities are the sum of non-Hispanic Black, Hispanics, Asian/Pacific Islander, Alaskan Native/American Indian, and other race/ethnicity groups that are not non-Hispanic White.)

| **ID** | **County Name** | **Non-Hispanic White** | **Non-Hispanic Black** | **Hispanic** | **Asian/Pacific Islander, Alaskan Native/American Indian** | **Minorities** |
| --- | --- | --- | --- | --- | --- | --- |
| 1 | Bronx, NY | 6.4 | 37.26 | 53.53 | 1.5 | 93.6 |
| 2 | Bronx, NY | 35.09 | 29.24 | 31.44 | 2.36 | 64.91 |
| 3 | Bronx, NY | 4.5 | 26.14 | 67.07 | 1.4 | 95.5 |
| 4 | Bronx, NY | 20.23 | 32.03 | 42.54 | 3.19 | 79.77 |
| 5 | Bronx, NY | 43.83 | 21.42 | 31.75 | 1.4 | 56.17 |
| 6 | Bronx, NY | 14.99 | 35.11 | 44.01 | 4.64 | 85.01 |
| 7 | Bronx, NY | 11.68 | 31.08 | 54.67 | 1.33 | 88.32 |
| 8 | Bronx, NY | 16.67 | 16.67 | 16.67 | 33.33 | 83.33 |
| 9 | Kings, NY | 76.43 | 10.37 | 5.07 | 2.47 | 23.57 |
| 10 | Kings, NY | 13.69 | 60.85 | 19.87 | 4.36 | 86.31 |
| 11 | Kings, NY | 37.9 | 38.83 | 18.91 | 1.69 | 62.1 |
| 12 | Kings, NY | 72.54 | 7.33 | 9.27 | 6.92 | 27.46 |
| 13 | Kings, NY | 68.71 | 11.83 | 8.85 | 5.49 | 31.29 |
| 14 | Kings, NY | 15.52 | 69.74 | 9.87 | 1.2 | 84.48 |
| 15 | Kings, NY | 5.52 | 78.64 | 8.86 | 1.34 | 94.48 |
| 16 | Kings, NY | 30.06 | 15.63 | 49.53 | 3.95 | 69.94 |
| 17 | Kings, NY | 17.3 | 59.17 | 18.72 | 1.8 | 82.7 |
| 18 | Kings, NY | 50.98 | 28.75 | 15.16 | 2.22 | 49.02 |
| 19 | Kings, NY | 59.42 | 6.98 | 22.94 | 9.47 | 40.58 |
| 20 | Kings, NY | 13.87 | 70.51 | 8.81 | 2.17 | 86.13 |
| 21 | Kings, NY | 11.34 | 32.36 | 53.48 | 2.08 | 88.66 |
| 22 | Kings, NY | 9.94 | 76.2 | 9.87 | 1.19 | 90.06 |
| 23 | Nassau, NY | 62.06 | 24.89 | 8.35 | 3.89 | 37.94 |
| 24 | Nassau, NY | 80.19 | 9.14 | 5.03 | 3.78 | 19.81 |
| 25 | Nassau, NY | 83.12 | 9.68 | 4.23 | 2.34 | 16.88 |
| 26 | Nassau, NY | 88.79 | 4.39 | 3.88 | 2.41 | 11.21 |
| 27 | Nassau, NY | 91.09 | 3.28 | 3.19 | 1.95 | 8.91 |
| 28 | Nassau, NY | 83.43 | 9.22 | 5.21 | 1.04 | 16.57 |
| 29 | Nassau, NY | 85.16 | 6.27 | 6.01 | 1.19 | 14.84 |
| 30 | Nassau, NY | 71.76 | 20.59 | 5.18 | 1.2 | 28.24 |
| 31 | Nassau, NY | 91.75 | 2.66 | 2.6 | 2.48 | 8.25 |
| 32 | Nassau, NY | 88.73 | 5.79 | 3.47 | 1.51 | 11.27 |
| 33 | Nassau, NY | 58.83 | 30.23 | 5.78 | 2.63 | 41.17 |
| 34 | New York, NY | 59.65 | 15.82 | 18.75 | 3.89 | 40.35 |
| 35 | New York, NY | 46.13 | 25.69 | 23.66 | 2.81 | 53.87 |
| 36 | New York, NY | 35.51 | 11.14 | 15.1 | 37.17 | 64.49 |
| 37 | New York, NY | 49.45 | 16.16 | 26.26 | 6.07 | 50.55 |
| 38 | New York, NY | 64.07 | 11.7 | 19.6 | 2.92 | 35.93 |
| 39 | New York, NY | 68.8 | 13.57 | 13.04 | 2.84 | 31.2 |
| 40 | New York, NY | 84.88 | 5.73 | 5.11 | 3.44 | 15.12 |
| 41 | New York, NY | 57.75 | 15.85 | 18.09 | 6.06 | 42.25 |
| 42 | New York, NY | 12.79 | 30.57 | 52.54 | 3.34 | 87.21 |
| 43 | New York, NY | 28.97 | 23.31 | 36.06 | 10.52 | 71.03 |
| 44 | New York, NY | 78.31 | 7.02 | 8.34 | 4.08 | 21.69 |
| 45 | New York, NY | 4.62 | 73.76 | 18.95 | 1.99 | 95.38 |
| 46 | New York, NY | 87.08 | 5.11 | 4.61 | 1.65 | 12.92 |
| 47 | New York, NY | 58.04 | 15.75 | 16.97 | 7.65 | 41.96 |
| 48 | New York, NY | 5.8 | 64.5 | 27.99 | 1.32 | 94.2 |
| 49 | Queens, NY | 54.97 | 28.62 | 12.3 | 1.47 | 45.03 |
| 50 | Queens, NY | 30.44 | 32.72 | 24.08 | 7.57 | 69.56 |
| 51 | Queens, NY | 57.39 | 11.91 | 12.02 | 15.8 | 42.61 |
| 52 | Queens, NY | 34.31 | 11.2 | 34.82 | 16.7 | 65.69 |
| 53 | Queens, NY | 51.27 | 12.81 | 15.9 | 18.62 | 48.73 |
| 54 | Queens, NY | 69.49 | 16.55 | 6.36 | 4.88 | 30.51 |
| 55 | Queens, NY | 16.16 | 49.29 | 14.87 | 12.76 | 83.84 |
| 56 | Queens, NY | 61.68 | 12.29 | 16.62 | 6.35 | 38.32 |
| 57 | Queens, NY | 49.7 | 32.34 | 13.07 | 1.97 | 50.3 |
| 58 | Richmond, NY | 68.11 | 14.28 | 12.52 | 4.24 | 31.89 |
| 59 | Richmond, NY | 83.85 | 5.09 | 6.46 | 2.2 | 16.15 |
| 60 | Rockland, NY | 79.93 | 9.86 | 6.76 | 2.88 | 20.07 |
| 61 | Rockland, NY | 81.97 | 9.59 | 5.38 | 2.36 | 18.03 |
| 62 | Rockland, NY | 87.84 | 5.2 | 4.46 | 1.88 | 12.16 |
| 63 | Suffolk, NY | 73.88 | 11.59 | 12.51 | 1.06 | 26.12 |
| 64 | Suffolk, NY | 90.26 | 4.54 | 3.16 | 1.63 | 9.74 |
| 65 | Suffolk, NY | 95.16 | 2.21 | 1.32 | 0.5 | 4.84 |
| 66 | Suffolk, NY | 88.91 | 6.75 | 2.71 | 0.63 | 11.09 |
| 67 | Suffolk, NY | 85.96 | 6.19 | 6.02 | 1.39 | 14.04 |
| 68 | Suffolk, NY | 91.96 | 2.68 | 3.64 | 0.85 | 8.04 |
| 69 | Suffolk, NY | 90.95 | 3.19 | 3.96 | 1.46 | 9.05 |
| 70 | Suffolk, NY | 82.92 | 9.08 | 5.93 | 1.08 | 17.08 |
| 71 | Suffolk, NY | 91.79 | 4.25 | 1.87 | 0.99 | 8.21 |
| 72 | Suffolk, NY | 87.01 | 5.1 | 4.93 | 1.75 | 12.99 |
| 73 | Suffolk, NY | 92.6 | 2.24 | 3.28 | 1.44 | 7.4 |
| 74 | Westchester, NY | 51.36 | 21.34 | 23.05 | 3.2 | 48.64 |
| 75 | Westchester, NY | 82.36 | 9.72 | 5.26 | 2.07 | 17.64 |
| 76 | Westchester, NY | 31.14 | 58.93 | 6.01 | 1.43 | 68.86 |
| 77 | Westchester, NY | 92.38 | 2.41 | 2.94 | 1.73 | 7.62 |
| 78 | Westchester, NY | 68.76 | 21.86 | 6.74 | 2.04 | 31.24 |
| 79 | Westchester, NY | 73.41 | 12.44 | 10.74 | 2.65 | 26.59 |
| 80 | Westchester, NY | 73.14 | 14.1 | 8.88 | 3.08 | 26.86 |
| 81 | Westchester, NY | 82.72 | 7.37 | 7.14 | 2.23 | 17.28 |
| 82 | Westchester, NY | 85.56 | 7.47 | 4.94 | 1.54 | 14.44 |
| 83 | Westchester, NY | 83.45 | 9.3 | 4.9 | 1.85 | 16.55 |
| 84 | Westchester, NY | 85.78 | 8.64 | 3.47 | 1.61 | 14.22 |
